# Metabolism-Guided LATTICE Radiotherapy in an Elderly Patient with Locally Advanced Head and Neck Cancer Treated with Curative Aim: A Case Report

**DOI:** 10.3390/reports8040213

**Published:** 2025-10-23

**Authors:** Giuseppe Iati’, Silvana Parisi, Giacomo Ferrantelli, Stefano Pergolizzi

**Affiliations:** 1 Department of Clinical and Experimental Medicine Radiation Oncology, University of Messina, 98122 Messina, Italy; 2 Department of Biomedical, Dental Science and Morphological and Functional Images, University of Messina, 98122 Messina, Italy; silvana.parisi@unime.it (S.P.); giacomo.ferrantelli@outlook.com (G.F.); stefano.pergolizzi@unime.it (S.P.)

**Keywords:** head and neck squamous cell carcinoma, spatially fractionated radiotherapy, elderly patient

## Abstract

**Background and clinical Significance**: The management of head and neck squamous cell carcinoma in elderly patients is a clinical scenario that is currently under debate. **Case Presentation**: Patients over 65 years old are particularly vulnerable, and the administration of curative oncological care is challenging. Furthermore, such treatment has the potential to be extremely toxic. Spatially fractionated radiation therapy (SFRT) is a radiotherapy modality that offers a promising approach for treating tumors. This method involves the delivery of a spatially modulated dose, resulting in highly non-uniform dose distributions. This leads to the generation of peaks and valleys of doses within a target volume. In this case study, a patient with an ulcerating lesion on the right cheek was treated with a two-phase radiotherapy regimen. The purpose of the first procedure was to stimulate the immunogenicity of the tumor microenvironment. In the second part of the procedure, standard fractionated irradiation was delivered with curative aim. **Conclusions**: The clinical response indicates that this combination of high-dose “localized” and low-dose irradiation can produce immunological effects with an acceptable toxicity profile.

## 1. Introduction and Clinical Significance

The epidemiology of head and neck squamous cell carcinoma (HNSCC) in elderly patients has changed over the past decade, with a rise in oropharyngeal cancers, likely due to HPV, and a fall in tobacco- and alcohol-related cancers in the oral cavity, larynx, and hypopharynx [1]. However, the limited data currently available may not adequately reflect the older patient population in current practice, underscoring the need for high-quality evidence to guide treatment strategies.

Elderly patients frequently present with multiple comorbidities and generally have a reduced capacity to tolerate intensive treatments [2]. In addition, studies have found that older adults do not gain significant benefit from treatment intensification, such as the addition of chemotherapy with radiation therapy (RT).

RT represents the most feasible treatment option for elderly patients with locally advanced (LA) head and neck carcinoma. The association with chemotherapy has been shown to have detrimental results in terms of toxicity [3]. Consequently, RT alone may lead to suboptimal therapeutic responses, highlighting the need for strategies to improve treatment efficacy. Hypofractionated regimens have been proposed as a potential approach to enhance the biological effectiveness of RT while reducing the overall treatment duration. Nevertheless, careful consideration must be given to the risk of increased toxicity associated with such schedules. In this context, spatially fractionated radiotherapy (SFRT) has emerged as a promising strategy, particularly for bulky tumors, with the potential to stimulate antitumor immune responses [4].

In this study, we present a case of advanced cheek cancer with infiltration and ulceration of the facial skin. The patient was treated using a metabolic-guided, non-geometric SFRT in combination with a normofractionated radiation regimen.

## 2. Case Presentation

An 82-year-old female patient presented with a baseline WHO performance status of 2 and an ulcerating lesion on the right cheek, which was fixed to the underlying planes and associated with pain and mucopurulent discharge. Confluent lymphadenopathies in the right laterocervical region at the IB level were observed.

**Figure 1:**
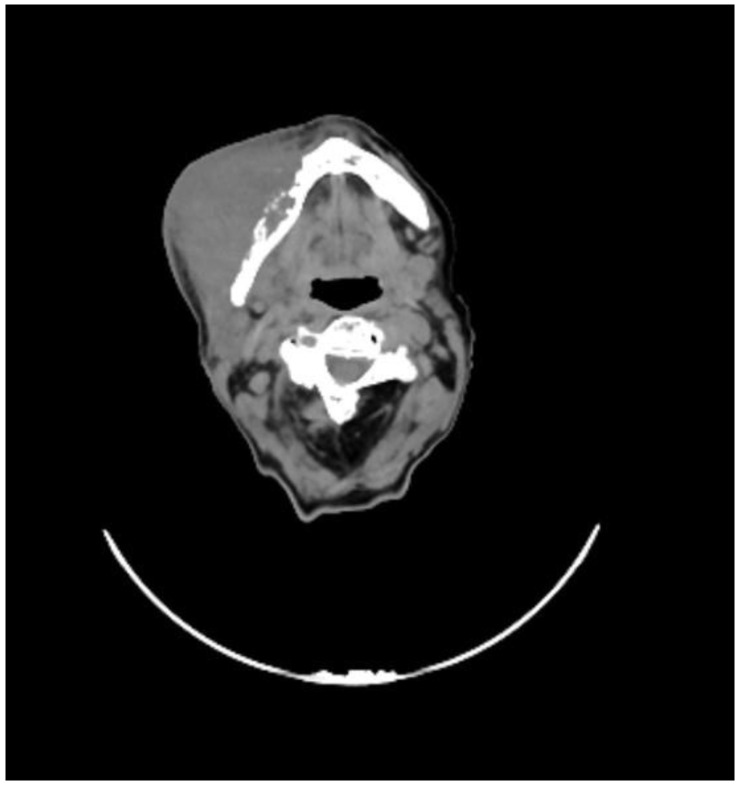
CT performed before the start of therapy, showing the extent of disease with infiltration of the skin and mandible.

She underwent a maxillofacial computed tomography (CT) scan, which revealed a lesion of the right genial mucosa with infiltration of the skin, buccinator muscle, and the cortex of the mandibular bone, affecting the alveolar canal, and it also revealed the presence of necrotic, colliquative, and confluent lymphadenopathies (Figure 1).

A positron emission tomography (PET) scan revealed intense uptake of the radiopharmaceutical of the known right cheek lesion (SUV max 12.20) and the presence of contextual hypodense areas of necrotic colliquative significance. The presence of lymphadenopathies in the ipsilateral submandibular level was evident.

An incisional biopsy of the lesion was performed, which revealed the presence of high-grade squamous cell carcinoma with a proliferating index (Ki67) of 50%.

The patient was staged as cT4N2a based on the 8th Edition of the American Joint Committee on Cancer (AJCC) Staging of Head and Neck Cancer.

After discussion of the case within the institutional multidisciplinary head and neck tumor board, the patient was considered eligible for exclusive radiotherapy. Written consent was acquired.

RT treatment was delivered using a combined approach: the patient first received a single session of LATTICE radiotherapy (LRT), followed 72 h later by conventional volumetric modulated arc radiotherapy (cVMAT). The patient was simulated in supine position with the arms along her sides and immobilized with a thermoplastic mask. The CT planning images were acquired on SOMATOM Sensation 16 Slice CT (Siemens Healthineers, Erlangen, Germany). Slices of 3 mm thickness were obtained from the vertex of the skull to the sternoclavicular junction level. Subsequently, simulation plan data were transferred to the Monaco treatment planning system (Elekta AB, Stockholm, Sweden). For LRT, CT scans for planning purposes were co-registered with an 18F-FDG-PET/CT scan.

**Figure 2:**
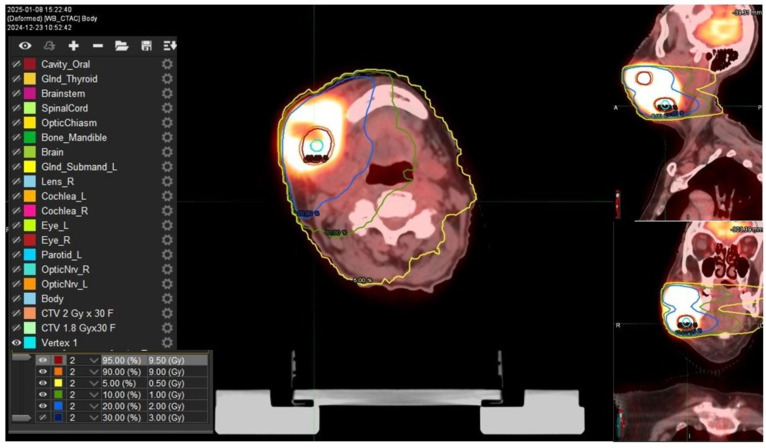
Isodose distribution of vertices utilized to create the dose inhomogeneity within the bulky disease (prescription dose 10 Gy in 1 fraction). Two vertices were applied at the interface between high-SUV and low-SUV areas. SFRT plan simulation was performed on PET-CT images coregistered with plan CT where there is a vertex (light blue), high isodose lines, the 95% (red line) and 90% (orange) of the prescription dose; and the low isodose line, delineating 5% (yellow line) of the prescription dose (0.5 Gy), 10% (green line) (1Gy), 20% (light blue line) (2 Gy), and 30% (dark blue line) 3 Gy.

A bulky gross tumor volume (B-GTV) was delineated, encompassing both the primary tumor mass and the gross lymphadenopathies identified on imaging studies. We chose a “metabolism-guided” lattice radiation treatment planning that we have previously described [5], delivering a spatially fractionated high radiation dose in two spherical deposits (vertices, Vs) within the bulky disease. Vs were allocated in a non-geometrical fashion between areas with different 18FDG/CT-PET metabolic activity levels (Figure 2). The dose delivered to the Vs was a single fraction of 10 Gy using a stereotactic volumetric modulated arc therapy (SVMAT) technique.

Three days later, the patient underwent a standard course of volumetric modulated arc radiotherapy (VMAT). At this stage, we defined a clinical target volume (CTV) expanding, isotropically, the B-GTV with a margin of 5 mm in all directions but taking into account the adjacent clinical structure as bones and skin; a dose of 60 Gy in 30 fractions was delivered. Furthermore, a low-risk clinical nodal volume (CNV) including the right Robbin levels from IA to IV was delineated and a dose of 54 Gy in 30 fractions was delivered.

The dosimetric analysis was based on the International Commission of Radiological Units and Measurements Reports 50 and 62. All plans were optimized to ensure target coverage and adherence to OAR dose constraints, referring to the Corsair parameters [6] as D mean, Dmax, and the percentage of volume receiving any dose (Vx). For CTV and CNV, we evaluated V95, V107, and Dmax; Dmean for the parotids and submandibular glands; Dmean for the oral cavity; Dmax, V50, and V42 for the mandible; Dmean for the constrictor muscles; and Dmax for spinal cord and for the esophagus.

**Figure 3:**
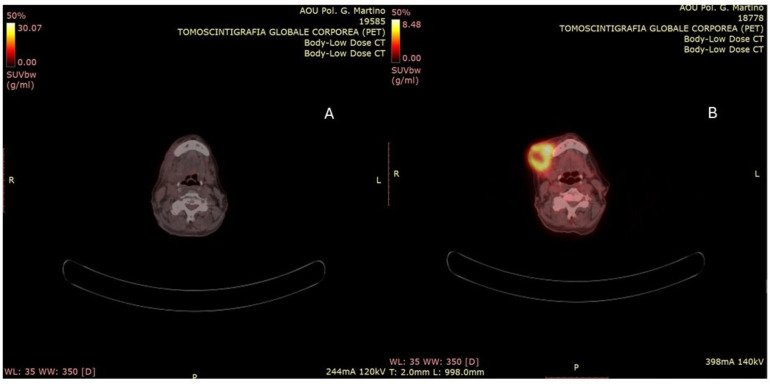
(**A**,**B**) PET-TC evaluation performed 90 days after the end of treatment. (**A**) The complete metabolic remission of the lesion compared to the staging PET CT (**B**).

**Figure 4:**
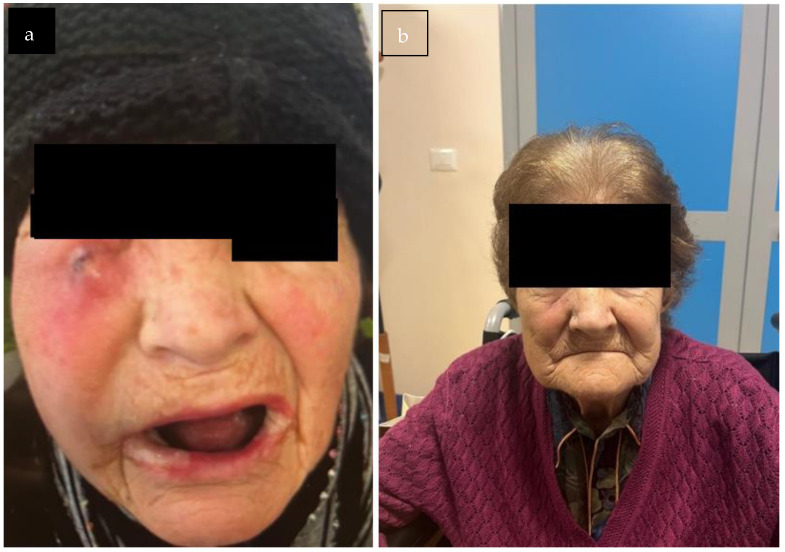
Cosmetic result, showing a complete response of the skin ulceration. (**a**) Patient at the initial presentation (September 2024). (**b**) Patient at the last follow-up (August 2025).

The V95 was 58 Gy for the GTV and 53 Gy for the CNV. The mean doses to the left parotid and submandibular glands were 10 Gy and 11 Gy, respectively. Mandibular V50 and V42 were 31% and 44%. The pharyngeal constrictor muscles received a mean dose of 26 Gy. Maximum doses to the spinal cord and esophagus were 30 Gy and 35 Gy, respectively. The patient completed the treatment without interruption. Ninety days after the conclusion of the treatment course, a PET-CT scan was performed, revealing a marked reduction in both the extent and intensity of radiotracer uptake when compared to the previous examination (Figure 3). From a clinical standpoint, there was clear evidence of complete resolution of the ulcerative skin lesion, with an excellent cosmetic outcome (Figure 4). Treatment-related toxicity was limited to grade 2 mucositis, which was resolved within two weeks through the administration of topical mouthwash containing an antifungal, corticosteroids, and lidocaine. At the last follow-up, 10 months after the completion of therapy, no signs of disease progression were observed.

## 3. Discussion

Most patients with HNSCC present LA disease (stages III–IVB) [7]. Although this stage is often potentially curable, achieving optimal control typically requires combined modality therapy. Each treatment modality presents unique challenges in older adults, who are more likely to have comorbidities that may preclude curative oncologic therapies, such as surgery. They are also more vulnerable to aspiration and often have compromised nutritional status, which can complicate treatment delivery. In patients deemed unsuitable for surgery, oncologists typically consider treatment intensification strategies, which have demonstrated improvements in overall survival and locoregional recurrence. These include concomitant chemotherapy (CRT), cetuximab, and altered fractionation of RT dosing using high-conformal-delivery techniques [8]. However, none of these strategies have shown any survival benefit in patients over 70.

These intensification strategies may be less effective at eradicating tumors in older adults due to the biological and microenvironmental differences characteristic of head and neck cancers in this age group. Moreover, the potential advantages of these intensive regimens are often burdened by treatment-related toxicities and by competing risks of non-cancer mortality [9]. Older adults with cancer, including HNSCC, are also more likely to have comorbidities and age-related pharmacodynamic changes that influence the metabolism of anticancer agents [10]. Advanced age is a strong predictor of increased late treatment-related toxicities in HNSCC, such as aspiration pneumonia, dysphagia, and gastrostomy tube dependence, as well as higher rates of non-cancer-associated mortality following CRT [11].

SFRT uses highly modulated spatial dose distributions to create alternating high-dose (“peaks”) and low-dose (“valleys”) regions within the tumor. This is achieved through shielding tools like blocks and multi-leaf collimators (MLCs) or advanced techniques like VMAT or SBRT, which enable the formation of sharp dose gradients across the treatment field. This treatment modality is of growing interest in radiation oncology, physics, and biology. There are clinical experiences showing that SFRT can achieve a high response and low toxicity in the treatment of refractory and bulky tumors [12].

There are several methods to reach this aim, such as GRID therapy, LRT, microbeam radiation therapy (MRT), minibeam radiation therapy (MBRT) and FLASH radiotherapy.

The first GRID therapy was performed by Mohiuddin et al. [13] in which an acerrobend block collimator (also called GRID collimator) was used and one fraction of a large nominal dose was given before starting conventional radiation therapy. The pencil beam obtained using this collimator has a regular geometric distribution inside the target, regardless of the metabolic tumor microenvironment heterogeneity. LRT is a 3D technique that uses SFRT to treat deep-seated tumors. It aims to deliver high-dose islands within the GTV and minimize the dose outside the GTV for better toxicity control [14]. MRT uses X-ray microbeams in a collimated array manner, with high dose rates and minimal beam divergence [15]. It is conceivable that by generating areas of low or high exposure, protective reservoirs of the tumor’s immune microenvironment can be created, thus preserving the anti-tumor immune responses essential for the success of radiotherapy [16]. SFRT has been observed to elicit exceptional clinical responses, despite its deviation from the noted radiobiological principles, enhancing both tumoral response and sparing normal tissues [17]**.**

In the present case study, the spherical deposits of high doses are allocated at the interface between regions exhibiting high metabolic activity, as determined by the PET scan, and those exhibiting low metabolic activity. Our hypothesis states that both the high-dose (“peak dose”) and low-dose (“valley dose”) regions of SFRT can promote an immunomodulatory effect against cancer.

Evidence from the literature suggests that high radiation doses cause cellular damage, which activates the innate (rapid) response, including the stimulation of antigen-presenting cells (APCs), such as dendritic cells, through the release of tumor-associated antigens [18]. Conversely, low-dose radiation (i.e., 0.5–2 Gy) has been observed to modulate the tumor microenvironment (TME), inducing a shift towards M1 macrophage polarization. Consequently, iNOS-positive M1 macrophages secrete chemokines that play a key role in the recruitment of effector T cells. Moreover, these macrophages enhance the tumor vasculature and inflammation, facilitating T-cell infiltration [19]. When delivered prior to conventional therapy, this treatment is hypothesized to enhance antitumor immunity by stimulating the immune system to recognize and attack cancer cells, thereby counteracting the immunosuppressive effects of the subsequent conventional radiotherapy course. This approach has been observed to promote a subsequent, slow adaptive response, which could counterbalance the immunosuppressive effect on locoregional lymph node germinal centers subjected to higher doses of radiotherapy used in standard treatments.

Several case reports and case series have documented that SFRT provides rapid symptom palliation in patients with advanced tumors and limited therapeutic options [20,21,22].

Currently, at least two ongoing studies, NCT06416007 and NCT07000162, aim to validate the efficacy of lattice radiation therapy in patients with advanced tumors. In addition to assessing clinical outcomes, these studies are expected to provide valuable insights into the immunomodulatory effects of such spatially fractionated regimens, both within the tumor microenvironment and systemically.

To the best of our knowledge, this is the first example in the literature that uses a metabolism-guided spatially fractionated radiotherapy (lattice-like approach) for curative purposes.

The study suggests that a heterogeneous dose distribution inside a B-GTV, localized in specific areas with different metabolisms, using the SFRT can promote an anti-tumor immune response. The evidence indicates that LRT may be a viable therapeutic option for cases that would otherwise be challenging to manage [23].

## 4. Conclusions

The present study reports on a patient with LA cheek cancer treated with SFRT with a curative aim. This treatment, obtained using a combination of high “localized” doses and low-dose irradiation, can produce immunological effects with an encouraging clinical result and an acceptable toxicity profile. Further studies are necessary to determine the validity of this therapeutic approach for head and neck carcinoma selective patients.

## Data Availability

The data presented in this study are available upon request from the corresponding author.

## Abbreviations

The following abbreviations are used in this manuscript:

HNSCC	Head and neck squamous cell carcinoma
RT	Radiotherapy
LA	Locally advanced
SFRT	Spatially fractionated radiotherapy
CT	Computed tomography
PET	Positron emission tomography
AJCC	American Joint Committee on Cancer
LRT	LATTICE radiotherapy
cVMAT	Conventional volumetric modulated arc radiotherapy
Vs	Vertices
SVMAT	Stereotactic volumetric modulated arc therapy
B-GTV	Bulky gross tumor volume
Vx	Percentage of volume receiving any dose
CTV	Clinical target volume
CNV	Clinical nodal volume
Dmax	Maximum of dose
Dmean	Mean dose
CRT	Chemo-radiotherapy
GRT	GRID radiotherapy
MRT	Microbeam radiation therapy
MBRT	Minibeam radiation therapy
APC	Antigen-presenting cells
TME	Tumor microenvironment

## References

[1] Zumsteg Z.S., Cook-Wiens G., Yoshida E., Shiao S.L., Lee N.Y., Mita A., Jeon C., Goodman M.T., Ho S.A. (2016). Incidence of Oropharyngeal Cancer Among Elderly Patients in the United States. JAMA Oncol..

[2] Machtay M., Moughan J., Trotti A., Garden A.S., Weber R.S., Cooper J.S., Forastiere A., Ang K.K. (2008). Factors associated with severe late toxicity after concurrent chemoradiation for locally advanced head and neck cancer: An RTOG analysis. J. Clin. Oncol..

[3] Haehl E., Rühle A., David H., Kalckreuth T., Sprave T., Stoian R., Becker C., Knopf A., Grosu A.L., Nicolay N.H. (2020). Radiotherapy for geriatric head-and-neck cancer patients: What is the value of standard treatment in the elderly?. Radiat. Oncol..

[4] Yan W., Khan M.K., Wu X., Simone C.B., Fan J., Gressen E., Zhang X., Limoli C.L., Bahig H., Tubin S. (2019). Spatially fractionated radiation therapy: History, present and the future. Clin. Transl. Radiat. Oncol..

[5] Ferini G., Parisi S., Lillo S., Viola A., Minutoli F., Critelli P., Valenti V., Illari S.I., Brogna A., Umana G.E. (2022). Impressive Results after “Metabolism-Guided” Lattice Irradiation in Patients Submitted to Palliative Radiation Therapy: Preliminary Results of LATTICE_01 Multicenter Study. Cancers.

[6] Bisello S., Cilla S., Benini A., Cardano R., Nguyen N.P., Deodato F., Macchia G., Buwenge M., Cammelli S., Wondemagegnehu T. (2022). Dose-Volume Constraints fOr oRganS at risk In Radiotherapy (CORSAIR): An “All-in-One” Multicenter-Multidisciplinary Practical Summary. Curr. Oncol..

[7] Maggiore R., Zumsteg Z.S., BrintzenhofeSzoc K., Trevino K.M., Gajra A., Korc-Grodzicki B., Epstein J.B., Bond S.M., Parker I., Kish J.A. (2017). CARG-HNC Study Group. The Older Adult with Locoregionally Advanced Head and Neck Squamous Cell Carcinoma: Knowledge Gaps and Future Direction in Assessment and Treatment. Int. J. Radiat. Oncol. Biol. Phys..

[8] Pontoriero A., Iatì G., Aiello D., Pergolizzi S. (2016). Stereotactic Radiotherapy in the Retreatment of Recurrent Cervical Cancers, Assessment of Toxicity, and Treatment Response: Initial Results and Literature Review. Technol. Cancer Res. Treat..

[9] Michal S.A., Adelstein D.J., Rybicki L.A., Rodriguez C.P., Saxton J.P., Wood B.G., Scharpf J., Ives D.I. (2012). Multi-agent concurrent chemoradiotherapy for locally advanced head and neck squamous cell cancer in the elderly. Head Neck.

[10] Bernardi D., Barzan L., Franchin G., Cinelli R., Balestreri L., Tirelli U., Vaccher E. (2005). Treatment of head and neck cancer in elderly patients: State of the art and guidelines. Crit. Rev. Oncol. Hematol..

[11] Argiris A., Brockstein B.E., Haraf D.J., Stenson K.M., Mittal B.B., Kies M.S., Rosen F.R., Jovanovic B., Vokes E.E. (2004). Competing causes of death and second primary tumors in patients with locoregionally advanced head and neck cancer treated with chemoradiotherapy. Clin. Cancer. Res..

[12] Zhang H., Wu X. (2024). Which Modality of SFRT Should be Considered First for Bulky Tumor Radiation Therapy, GRID or LATTICE?. Semin. Radiat. Oncol..

[13] Mohiuddin M., Fujita M., Regine W.F., Megooni A.S., Ibbott G.S., Ahmed M.M. (1999). High-dose spatially fractionated radiation (GRID): A new paradigm in the management of advanced cancers. Int. J. Radiat. Oncol. Biol. Phys..

[14] Wu X., Perez N.C., Zheng Y., Li X., Jiang L., Amendola B.E., Xu B., Mayr N.A., Lu J.J., Hatoum G.F. (2020). The Technical and Clinical Implementation of LATTICE Radiation Therapy (LRT). Radiat. Res..

[15] Meyer J., Eley J., Schmid T.E., Combs S.E., Dendale R., Prezado Y. (2019). Spatially fractionated proton minibeams. Br. J. Radiol..

[16] Bekker R.A., Obertopp N., Redler G., Penagaricano J., Caudell J.J., Yamoah K., Pilon-Thomas S., Moros E.G., Enderling H. (2024). Spatially fractionated GRID radiation potentiates immune-mediated tumor control. Radiat. Oncol..

[17] McMillan M.T., Khan A.J., Powell S.N., Humm J., Deasy J.O., Haimovitz-Friedman A. (2024). Spatially Fractionated Radiotherapy in the Era of Immunotherapy. Semin. Radiat. Oncol..

[18] Carvalho H.A., Villar R.C. (2018). Radiotherapy and immune response: The systemic effects of a local treatment. Clinics.

[19] Herrera F.G., Ronet C., Ochoa de Olza M., Barras D., Crespo I., Andreatta M., Corria-Osorio J., Spill A., Benedetti F., Genolet R.G. (2022). Low-Dose Radiotherapy Reverses Tumor Immune Desertification and Resistance to Immunotherapy. Cancer Discov..

[20] Parisi S., Sciacca M., Critelli P., Ferrantelli G., Chillari F., Venuti V., Napoli C., Shteiwi I., Siragusa C., Brogna A. (2024). Lattice radiotherapy in inflammatory breast cancer: Report of a first case treated with curative aim. Radiat. Oncol. J..

[21] Xu P., Wang S., Zhou J., Yuan K., Wang X., Li L., Lang J., Lu S. (2024). Spatially fractionated radiotherapy (Lattice SFRT) in the palliative treatment of locally advanced bulky unresectable head and neck cancer. Clin. Transl. Radiat. Oncol..

[22] Studer G., Jeller D., Streller T., Huebner D., Glanzmann C. (2024). Time-Related Outcome Following Palliative Spatially Fractionated Stereotactic Radiation Therapy (Lattice) of Large Tumors—A Case Series. Adv. Radiat. Oncol..

[23] Ferini G., Valenti V., Tripoli A., Illari S.I., Molino L., Parisi S., Cacciola A., Lillo S., Giuffrida D., Pergolizzi S. (2021). Lattice or Oxygen-Guided Radiotherapy: What If They Converge? Possible Future Directions in the Era of Immunotherapy. Cancers.

